# *E. coli* aggregation and impaired cell division after terahertz irradiation

**DOI:** 10.1038/s41598-021-99665-3

**Published:** 2021-10-14

**Authors:** Sergey Peltek, Irina Meshcheryakova, Elena Kiseleva, Dmitry Oshchepkov, Alexei Rozanov, Danil Serdyukov, Evgeniy Demidov, Gennady Vasiliev, Nikolay Vinokurov, Alla Bryanskaya, Svetlana Bannikova, Vasiliy Popik, Tatyana Goryachkovskaya

**Affiliations:** 1 Laboratory of Molecular Biotechnologies of Federal Research Center Institute of Cytology and Genetics of the Siberian Branch of the Russian Academy of Sciences, 10 Lavrentiev Aven., Novosibirsk, Russia 630090; 2 Kurchatov Genomics Center of Federal Research Center Institute of Cytology and Genetics of the Siberian Branch of the Russian Academy of Sciences, 10 Lavrentiev Aven., Novosibirsk, Russia 630090; 3 Budker Institute of Nuclear Physics of the Siberian Branch of the Russian Academy of Sciences, 11 Lavrentiev Aven., Novosibirsk, Russia 630090

**Keywords:** Biophysics, Cell biology, Genetics, Microbiology, Molecular biology

## Abstract

In this study we demonstrated that exposure of *Escherichia coli* (*E. coli*) to terahertz (THz) radiation resulted in a change in the activities of the *tdcABCDEFGR* and *matA–F* genes (signs of cell aggregation), gene *yjjQ* (signs of suppression of cell motility), *dicABCF*, *FtsZ*, and *minCDE* genes (signs of suppression of cell division), *sfmACDHF* genes (signs of adhesin synthesis), *yjbEFGH* and *gfcA* genes (signs of cell envelope stabilization). Moreover, THz radiation induced *E. coli* csg operon genes of amyloid biosynthesis. Electron microscopy revealed that the irradiated bacteria underwent increased aggregation; 20% of them formed bundle-like structures consisting of two to four pili clumped together. This could be the result of changes in the adhesive properties of the pili. We also found aberrations in cell wall structure in the middle part of the bacterial cell; these aberrations impaired the cell at the initial stages of division and resulted in accumulation of long rod-like cells. Overall, THz radiation was shown to have adverse effects on bacterial populations resulting in cells with abnormal morphology.

## Introduction

THz radiation is most often regarded as electromagnetic waves corresponding to a frequency range of 10^11^ to 3 × 10^13^ Hz (wavelengths from 10 μm to 3 mm)^1^. The research field of electromagnetic radiation of the THz frequency range has rapidly advanced only in the last three decades owing to the lag in the development of THz sources and detectors in comparison with those for other frequency ranges of the electromagnetic spectrum. On the other hand, THz radiation from natural sources is almost completely absorbed by the atmosphere, and the evolution of organisms in the Earth biosphere has taken place in the almost complete absence of exposure to this type of radiation. Therefore, technogenic-origin THz radiation is a stressor for living systems, and the data on effects of this physical factor on living objects have been already obtained in studies on various biological entities^2–4^. It is noteworthy that these studies concern the nonthermal THz effect that is not caused by the heating of the exposed objects. In contrast to the current active development of THz technologies in many areas, the number of relevant biological studies remains small overall. This is especially true of the effect of THz radiation on the genetic apparatus: such works have been mainly implemented only during the last decade^3,4^. It is genetic and other biological studies on THz topics that turn out to be important in an adequate assessment of the biosafety of the technologies based on THz radiation.

*Escherichia coli* is a classic research object in biology; it is an easy-to-use prokaryotic model organism, which has already been used by us earlier in the studies on changes in the cell genetic apparatus under THz irradiation^5–9^. In the present study, we intended to obtain new information on the non-thermal effects of THz electromagnetic waves on bacteria at the molecular-genetic and cellular levels. As a result, after THz irradiation of *E. coli* cells, using high-throughput RNA sequencing (RNA-seq), we identified a set of differentially expressed genes and found genes that show changes in expression in a coordinated manner after the irradiation. Next, by bioinformatics analysis, we determined gene networks associated with changes in pili adhesion, with cell aggregation, and with assembly of the septal ring. Electron microscopy methods revealed that THz irradiation causes cell aggregation and pilus bundling with the formation of bundle-like structures (filaments) composed of one, two, or three docked rod components of pili as well as defects of cell envelope invaginations at an early stage of cell division resulting in cell elongation.

## Results

On the basis of the RNA-seq data, differences in the concentrations of RNAs were found between the irradiated and control cells. By means of these data, we identified genes whose expression changed after the irradiation. For subsequent analyses, 546 genes were chosen whose expression increased more than fourfold (log_2_ fold change ≥ 2, padj < 0.05) and 195 genes with a decrease in expression more than twofold (log2 fold change ≤  − 1, padj < 0.05); the list of differentially expressed genes (padj < 0.05) is given in the Supplementary Table S5. Raw sequence reads are available in the NCBI Sequence Read Archive (SRA; submission PRJNA648263).

### The search for functional relations among the differentially expressed genes

This procedure was carried out in the *E. coli* K12 MG1655 genome via the STRING database (Search Tool for the Retrieval of Interacting Genes/Proteins; string-db.org), version 10.5, with all available sources for the search for interactions and minimal probability of 0.4 for the existence of a relation between two genes in the Kyoto Encyclopedia of Genes and Genomes (KEGG) database (string-db.org). Networks constructed for the genes upregulated and downregulated by the THz irradiation were found to be highly linked (protein–protein interaction enrichment p-value < 1.0e^−16^).

The set of upregulated genes was found to be enriched with cellular component “pilus” (GO.0009289), molecular functions of fimbrial porins (GO.0015473), and biological processes associated with the organization and assembly of pili (GO.0043711 and GO.0009297) as well as cell adhesion (GO.0007155). The data on functional enrichment are provided in Supplementary Table S1. Functional examination of the proteins related to the GO.0007155 term “cell adhesion” suggested that the adhesion is also mediated by the proteins of pili. The set of downregulated genes turned out to be enriched with cytoplasmic genes (GO.0044444), genes of the respiratory chain (GO.0070469) and of ribosomes (GO.0005840), and with processes of aerobic respiration (GO.0009060) and translation (GO.0006412). Some of the most enriched terms are listed in Supplementary Table S2.

Manual analysis of the complete gene list revealed differential expression of the genes responsible for colanic acid biosynthesis, genes of Curli-type amyloid fimbriae and of genes that control cell division. Table 1 lists groups of genes whose products are related to the assembly and organization of various types of pili as well as genes responsible for colanic acid biosynthesis and control of cell division. The genes with expression changes more than twofold are given.

**Table 1:** Terahertz radiation–induced changes in the expression of the genes responsible for the biosynthesis of pili and of colanic acid as well as the genes that control cell division.

Genes responsive to THz		FoldChange	Benjamini–Hochberg adjusted p-value
**Genes of transcription factors related to the regulation of cell aggregation processes**			
Threonine dehydratase operon activator protein	*tdcR*	12.1	1.13E−11
HTH-type transcriptional regulator EcpR	*matA*	8.9	3.48E−47
HTH-type transcriptional regulator YdeO	*ydeO*	8.5	2.27E−33
HTH-type transcriptional regulator DctR	*yhiF*	8	3.87E−19
Putative transcription factor YjjQ	*yjjQ*	7.7	1.58E−19
**Genes of chaperone-usher fimbria biogenesis**			
*elf*ADCG fimbrial operon	*elfA (*ycbQ)	4.2	2.24E−09
	*elfC (*ycbS)	4.8	2.51E−18
	*elfD (*ycbR)	5.9	7.78E−15
*sfm*ACDHF fimbrial operon	*sfmA*	8.1	1.04E−12
	*sfmC*	11.8	1.68E−27
	*sfmD*	7	3.73E−29
	sfmH	10.1	8.29E−24
	sfmF	15	1.75E−13
*yad*CKLM-htrE-yadVN fimbrial operon	*yadC*	8.3	2.34E−48
	*yadK*	9.1	1.10E−26
	*yadL*	5	2.69E−14
	*yadM*	7.8	2.87E−23
	*htrE*	9	2.03E−40
	*ecpD*	7.8	1.50E−11
	*yadN*	5.8	5.16E−08
*yeh*ABCD fimbrial operon	*yehB*	5.3	9.04E−28
	*yehC*	8.1	1.62E−23
	*yehD*	2.6	2.72E−06
*yfc*OPQRSUV fimbrial operon	*yfcS*	4.5	1.07E−06
	*yfcQ*	7.6	1.84E−08
	*yfcR*	4.3	1.92E−05
	*yfcV*	7.7	2.29E−11
*yra*HIJK fimbrial operon	*yraI*	7.5	6.11E−21
	*yraJ*	5.4	1.02E−26
	*yraH*	4.2	3.73E−11
Uncharacterized fimbrial chaperone	*ybgP*	4.6	8.28E−06
	*yqiH*	6.9	7.53E−31
	*yhcA*	9.4	2.72E−31
Uncharacterized fimbrial-like protein	*ybgD*	4.1	7.74E−12
	*ybgO*	7.8	2.31E−15
	*yqiI*	7.2	1.29E−36
	*ydeR*	8.7	5.52E−21
Uncharacterized outer membrane usher protein	*yhcD*	5.1	1.89E−32
**Genes of curli amyloid fibril biogenesis**			
*csgBAC* operon	*csgA*	4.4	9.51E−09
	*csgB*	5.1	1.16E−07
	*csgC*	9.2	4.65E−17
*scgDEFG* operon	*csgF*	3.7	4.29E−06
	*csgE*	3.3	2.99E−09
**Genes of colanic acid biosynthesis**			
Colanic acid biosynthesis	*wcaA*	3.9	0.000926
	*wcaC*	7.2	1.24E−05
	*wcaD*	8.3	1.73E−29
	*wcaE*	7.3	2.63E−08
	*wcaF*	6.4	0.000421
	*wcaJ*	3	0.003229
	*wcaL*	2.5	0.001523
	*wcaM*	4.6	5.43E−15
	*wcaK*	3	0.000160
	*wzc*	4.1	5.88E−09
	*wza*	6.4	1.19E−09
	*cpsB*	3.7	3.78E−06
	*cpsG*	2.7	0.004211
	*gmd*	3.7	0.000239
	*fcl*	3.5	0.006570
	*ypdI*	9.3	3.93E−11
	*nudD*	9.1	0.0008488
Biofilm formation	*ydeO*	8.5	2.27E−33
	*yddL*	8.7	2.46E−10
**Genes related to cell division**			
Z-ring assembly	*ftsA*	0.6	3.79E−07
	*ftsZ*	0.5	2.67E−09
	*ftsN*	0.6	0.000344
	*ftsQ*	0.6	0.000134
	*zapA*	0.5	9.33E−08
	*zipA*	0.6	1.48E−05
Z-ring positioning	*minE*	0.5	5.97E−07
	*minC*	0.5	9.01E−08
	*minD*	0.5	7.97E−10
Cell shape determination	*mreB*	0.6	1.84E−06
Cell division inhibition	*kilR*	9.5	1.83E−13
	*dicF*	5.6	0.0254138
	*dicB*	19	2.31E−09

**Figure 1:**
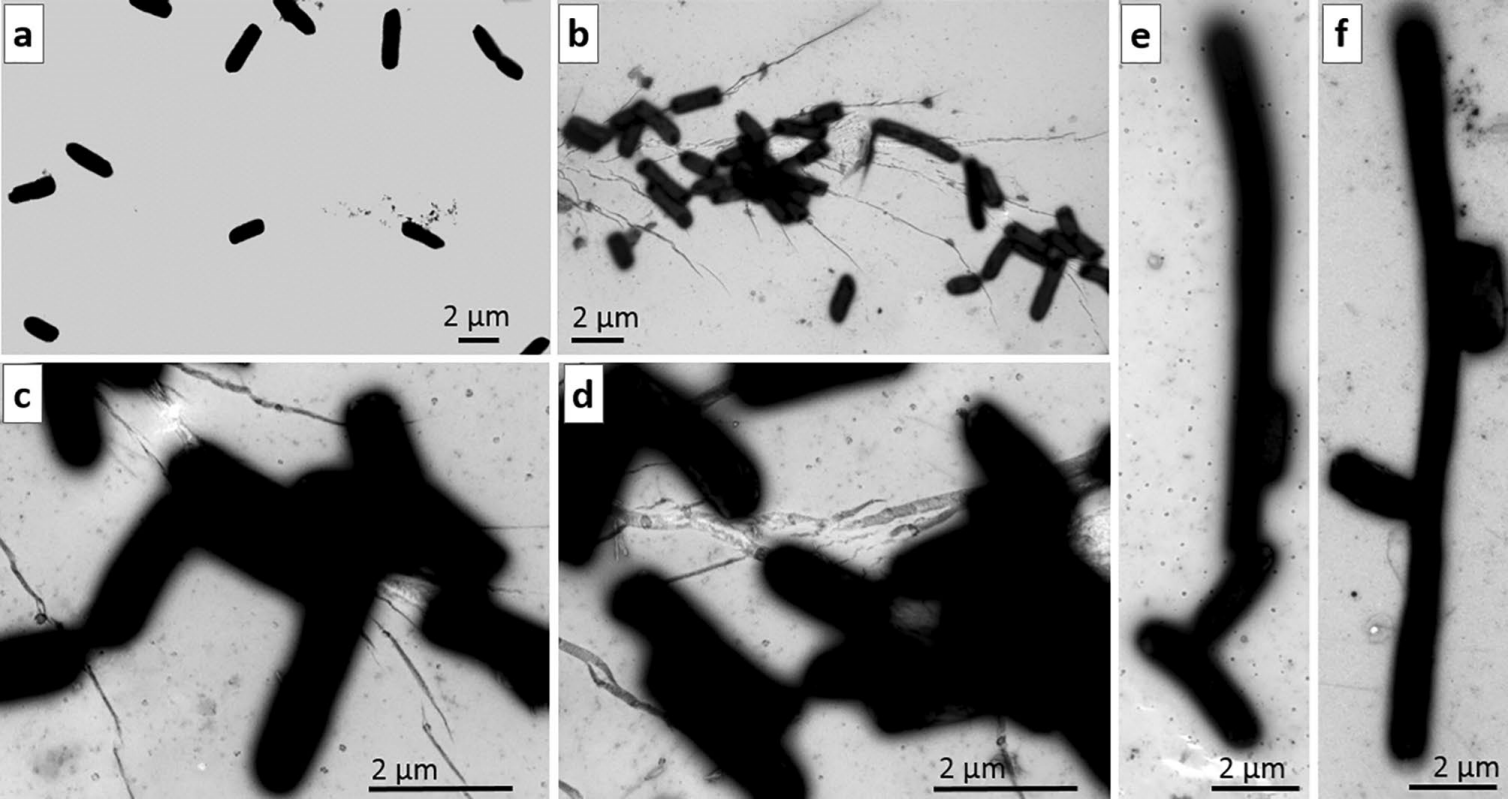
Aggregation of *E. coli* cells after treatment with THz radiation. (**a**) Stand-alone bacteria in the unirradiated sample. (**b**) Agglomeration of aggregated bacteria after the irradiation. (**c,d**) Magnified parts of panel (**b**). (**b–f**) Examples of aggregation of short and elongated (filamentous) bacteria. (**b–d**) Bacteria aggregate via side surfaces. (**e,f**) Bacteria aggregate through apical regions. The scale bar is 2 µm. Negative contrast.

**Figure 2:**
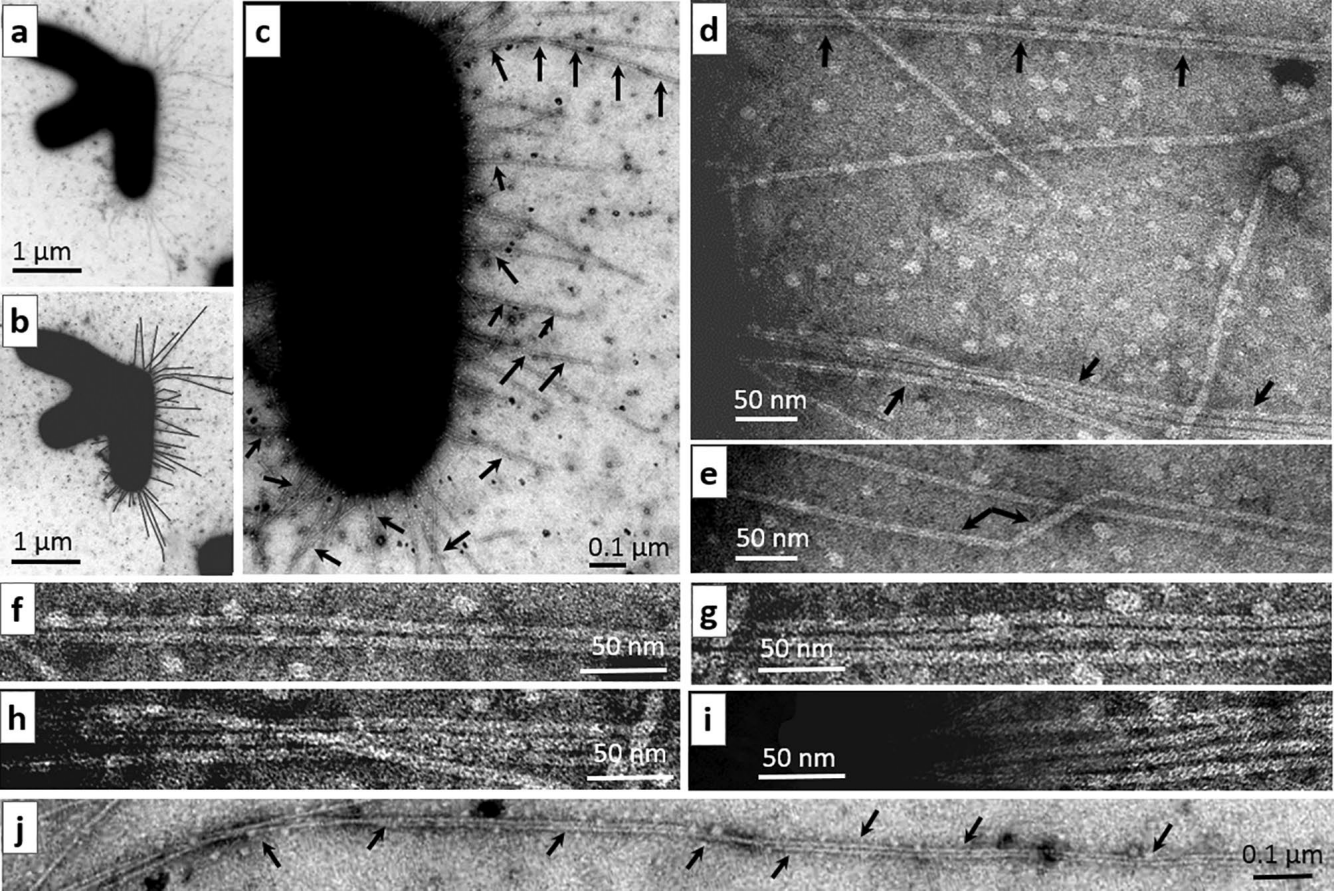
Multiple bundling of type 1 pili on *E. coli* cells after the THz irradiation. (**a**) Three aggregated bacterial cells are shown, one of which carries numerous type 1 pili: these pili have a diameter of 7.0–7.5 nm and vary in length from 0.1 to 2 µm, with maximal length in rare case reaching 3 µm. (**b**) The copy of (**a**) in which pili are indicated by black lines. (**c**) A magnified part of (**a**) with numerous examples of bundled pili (indicated by arrows) at low magnification. (**d**) A magnified part of (**a**) in which the arrows indicate two-pilus bundles. (**e**) Changes in the diameter of a pilus, indicated by interconnected arrows. (**f–i**) Examples of 2-, 3-, and 4-pilus bundles. (**j**) Adhesion of two pili throughout their considerable length. The scale bar is 1 µm in (**a,b**), 0.1 µm in (**c,j**), and 50 nm in (**d–j**).

Electron microscopy revealed that in the control samples, *E. coli* cells typically did not form large aggregates and did not manifest signs of their tight direct contacts (Fig. 1a), although bacteria connected via sex F pili were detectable. Analysis of the irradiated bacteria uncovered their multiple widespread contacts (Fig. 1b–f) with the participation of pili (Fig. 2a, Supplementary Fig. S4) and without them (Fig. 1b–f). Most bacteria were in contact through side surfaces along their long axis (Fig. 1b–d, Supplementary Fig. S4), but there were cases of adhesion between bacteria via their apical regions (Figs. 1c,e,f, 2a).

Next, we electron microscopically evaluated specific features of the organization of pili. According to the data from ultrastructural analysis of the bacteria in the control culture (Supplementary Fig. S3) and irradiated culture (Fig. 2, Supplementary Fig. S4), the cells carried type 1 pili. In the irradiated samples, there were bacteria with pili in close contact with bacteria without pili (Fig. 2a, Supplementary Fig. S4). In contrast to the control samples, in the irradiated ones, we revealed noticeable mutual bundling of two (Fig. 2c–f,j; Supplementary Fig. S4), and sometimes three (Fig. 2g,h) and four (Fig. 2i) pili giving rise to multilayer aggregates (bundles). The bundling of pili was often seen near the surface of the bacterial envelope (Fig. 2c,d, Supplementary Fig. S4) and could span a large distance, sometimes up to 3 µm (Fig. 2j). Two-pilus bundles were observed more often than the other versions (Fig. 2c, Supplementary Fig. S4). The diameter of most pili was stable, at 7 nm; however, in some regions, it reached 9 nm (Fig. 2e).

### Quantitative analysis of altered *E. coli* cells and pili in the groups before and after THz irradiation

The analysis was performed on 50 *E. coli* cells randomly selected from each of the two groups (before and after THz treatment) in two independent experiments (Exp 1 and Exp 2). The numbers of cells with normal and bundled pili, the numbers of doubled, triple, and quadruple pili per cell, and the number of elongated cells exceeding 5 μm in length were determined. The results of the quantitative analysis are shown in Table 2.

**Table 2:** Influence of THz irradiation on the numerical characteristics of an *E. coli* cell population.

Exp	Cells (N)	Cells (N) with pili	Pili (N max) per one cell	Cells -N1 with double pili (N2)	Cells with triple pili (N)	Cells with quadruple pili (N)	Long cell (N)
1-cont	50	12	41	4 (2)	0	0	2
1-THz	50	9	43	6 (18)	3	0	8
2-cont	50	13	42	6 (2)	0	0	2
2-THz	50	12	36	8 (20)	2	1	7

Determination of the number of bacterial cells with pili, the total number of pili per cell, the number of bacteria with clumped pili (two-pilus, three-pilus, and four-pilus bundles), as well as the number of elongated (more than 5 μm) bacterial cells was performed on 50 *E. coli* cells randomly selected from each of the following four groups: 1-cont and 2-cont, nonirradiated cells; 1-THz and 2-THz, irradiated cells. The analysis was carried out by means of an electron microscope on preparations obtained by the negative contrast method.

*N* the number of counted cells and pili.

The quantitative analysis of the features of structural organization and aggregation of *E. coli* cells before and after THz irradiation in two independently conducted experiments showed that the relative numbers of cells with pili were similar and ranged from 24 to 26% in the control and from 18 to 24% in the experimental group (Table 2). That the pili were not present on all bacterial cells is possibly related to the cell growth stage (associated with different phases of their cell cycle^10^). The number of pili per cell remained virtually unchanged after the irradiation, but the number of clumped pili per bacterial cell was 4.8% in the control and increased after the irradiation and varied from 41 to 55.5%. The relative number of cells with doubled pili (two-pilus bundles) increased from 40 in the control to 66.6% in the experimental group with respect to all cells with pili. Triple and quadruple pili (numbering 3 and 1 per cell, respectively) were detected only on irradiated bacterial cells. The obtained electron microscopy data in combination with the quantitative analysis indicated a stimulatory effect of THz irradiation on processes of *E. coli* cell aggregation and of atypical adhesion of *E. coli* pili*.*

**Figure 3:**
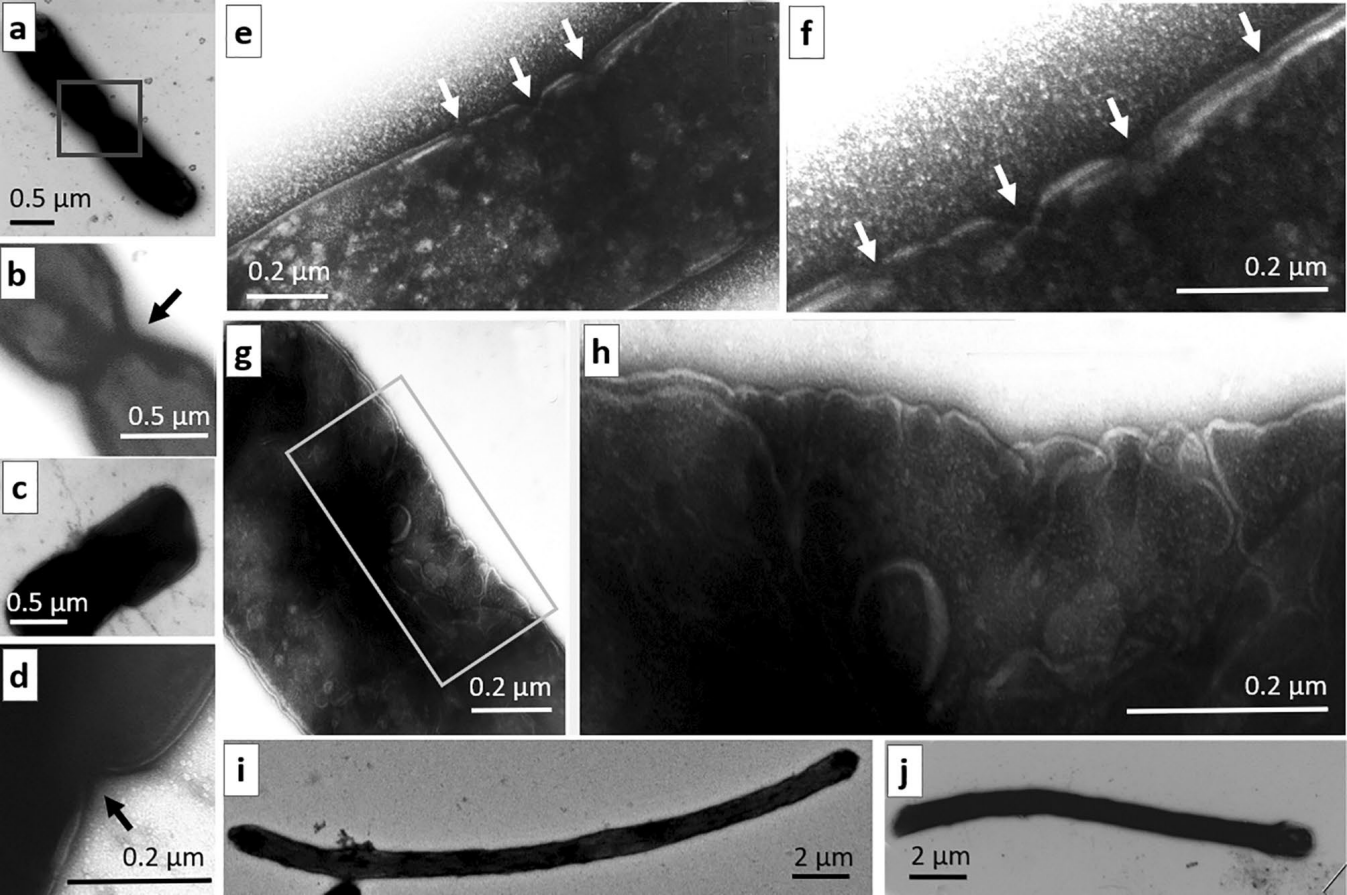
Disrupted structural organization of the envelope of dividing *E. coli* cells*,* as detected after exposure to THz radiation. (**a–d**) Examples of two dividing bacterial cells at low and high magnification with V-shaped invaginations in the envelope (indicated by arrows) in control. (**e,f**) Multiple V-shaped invaginations and breaches in the envelope of irradiated bacteria at low and high magnification (indicated by arrows). (**g,h**) Asymmetric unilateral formation of numerous folds in the envelope in the central part of an irradiated cell. The scale bar is 0.5 μm in (**a–c**); 0.2 μm in (**d–h**); and 2 μm in (**i,j**).

Electron microscopy methods applied to the irradiated culture revealed some defects in the envelope of dividing bacteria at the beginning of this process (Fig. 3). In the control samples, the bacterial envelope is composed of the outer and cytoplasmic membranes separated by periplasmatic space. Analyzing of the envelope of dividing bacteria at the initial stage of this process, we noted the emergence of a V-shaped symmetrical or ring-like invaginations of the envelope (Fig. 3a–d). In the irradiated bacteria, in the central area of the cells, occasionally, there were multiple V-shaped invaginations and breaches affecting both membranes of the bacterial envelope (Fig. 3e,f). There were also bacteria whose envelope looked like multiple rounded folds asymmetrically positioned in the middle part and separated by deep invaginations (Fig. 2g,h). Besides, the irradiated samples more often (1.6-fold) contained elongated bacteria (filamentous phenotype) with the length exceeding two to threefold the length of bacteria in the control samples and occasionally reaching 6 µm and more (Fig. 3i,j).

## Discussion

The typical behavior of bacteria under stressful conditions is the switch from planktonic growth to biofilm formation^11^. Colonization and biofilm formation begin with the adhesion of bacterial cells to available surfaces. During the transition to biofilm formation, bacteria decrease their motility and start to actively synthesize adhesins^12,13^.

According to our electron microscopic analysis, THz irradiation disrupts the invagination and breaches in the central region of the bacterial cell, thereby affecting both membranes of the bacterial envelope at the initial stages of cell division. To date, the key parameters of various stages of bacterial cell division have been thoroughly investigated by genetic, biochemical, and super-resolution imaging methods. During cell division, a complicated system is formed—the divisome—which includes a septal ring attached under the cell membrane (Z-ring) and encircling the cell in the middle; the divisome consists of bundles of laterally linked protofilaments resulting from the polymerization of the FtsZ protein and a set of auxiliary proteins^14,15^. Interruption of the synthesis of divisome proteins either in mutant cells or under the influence of stressful conditions can block cell division and cause elongation of bacteria or may initiate multiple processes of division randomly distributed along the cell, thereby resulting in the mini-cell phenotype^16^.

The process of cell division includes polymerization of a tubulin like protein into a ring, with subsequent formation of the septum with the help of proteins FtsZ, FtsN, FtsQ, ZapA, and ZipA^17,18^. Dysfunction of one of these proteins can not only initiate a cascade of negative events affecting the interaction between these proteins but also disrupt their connections with other proteins of the divisome that determine the progression of cytokinesis and the synthesis of proteins needed for the construction of the septum. We noticed that the mRNA expression of these five proteins is lower in the irradiated *E. coli* culture: FtsZ, a 0.5-fold change; FtsN, a 0.6-fold change; FtsQ, a 0.6-fold change; ZapA, a 0.5-fold change; and ZipA, a 0.6-fold change, Additionally, there was underexpression of a dynamic cytoskeletal protein (gene *mreB*). The expression of key inhibitors of cell division (*dicB* “division control B” and *dicF* “division control F”) after the THz irradiation substantially increased: 19- and fivefold, respectively. DicB inhibits cell division by interacting with and affecting the localization and activity of cell division proteins MinC and FtsZ^19^.

According to some research, disruptions of bacterial cell division arise during stoichiometric failure in the organization of the divisome and septal ring^20^. The emergence of numerous elongated (filamentous) bacteria, as seen here after THz irradiation (Figs. 1e,f, 3i,j) is similar to that among *ftsZ*-mutant bacteria, which form long chains consisting of unseparated cells^21,22^.

Additionally, our RNA-seq analysis of *E. coli* cells indicates that under THz irradiation, the expression of genes *minC*, *minD*, and *minE* in the irradiated cells decreases twofold as compared with the unirradiated group. The corresponding proteins MinC, MinD, and MinE assemble into a complex with FtsZ and are necessary for correct division of *E. coli* cells^22,23^. The expression of genes coding for proteins FtsZ and FtsA (homologs of tubulin and actin) decreases. It is known that both proteins function at initial stages of division of *E. coli* cells and are mutual regulators for dynamic assembly–disassembly of oligomers of each one^18^.

In our study, electron microscopy suggests that under THz irradiation, *E. coli* cells produce a large number of two-pilus bundles adhering via side surfaces. Moreover, in our experiments, we registered mutual adhesion of not only two but also three and four pili, with this phenomenon seen throughout their considerable length. These data may indicate increased adhesiveness of the pili, possibly as a consequence of alterations in the adhesive properties of protein constituents of pili. These observations were confirmed by the results of RNA-seq, which showed that under THz irradiation, there is overexpression of seven genes of transcription factors, five of which are associated with the regulation of processes of cell aggregation: *tdcR*, *matA*, *ydeO, yhiF* and *yjjQ*. Transcription factor TdcR controls the genes involved in the transport and metabolism of serine and threonine. Acylated lactones of homoserine in gram-negative bacteria serve as autoinducers of quorum sensing^24,25^. Transcription factor MatA activates the processes of *E. coli* transition from a planktonic to adherent state. The *mat* (*yag* or *ecp*) operon is composed of six genes, *matA–F*, where *matA* encodes a transcription factor^26,27^. The THz radiation also enhanced the expression of genes *matC* and *matB* from this operon. Under the conditions of planktonic growth, the *mat* operon in *E. coli* K12 is not expressed. In a paper by Lehti et al., they state that overexpression of the *matABCD* operon gives rise to biofilms at 20 °C^27^. Transcription factor MatA represses the *flhDC* operon, which governs the biosynthesis of bacterial flagella, motility, and chemotaxis of microorganisms^28^. Thus, through transcription factor MatA, THz radiation suppresses cell motility. The opposite actions of MatA on operons *mat* and *flhDC* may regulate the transition of *E. coli* from a planktonic to adherent state: flagellar biosynthesis is repressed, and the cell is getting ready for aggregation via the biosynthesis of a pilus and adhesive proteins. The flagellar biosynthesis is also repressed by transcription factor YjjQ (upregulated 7.7-fold by THz radiation)^29^.

Transcription factor YdeO activates genes of resistance to acid and anaerobic conditions and is induced by UV radiation^30^. Overexpression of YdeO results in *dctR* upregulation. Overexpression of DctR (*yhiF)* affects cellular morphology and causes filamentous biofilm formation^31^. After the THz irradiation, the expression of transcription factor gene *yhiF* increased eightfold.

Bioinformatic analysis of the RNA sequencing results by means of STRING revealed biological processes GO:0043711 (pilus organization), GO:0009297 (pilus assembly), GO.0009289 (pilus), and other closely related processes, for example, GO.0007155 (cell adhesion).

Our RNA-seq findings suggest that the exposure to THz radiation induce the expression of six cryptic operons encode putative chaperone-usher fimbriae promote adhesion *E. coli* K-12 to different surfaces^32^. Genes of operon *sfmACDHF* were upregulated more than sevenfold, and the expression of four genes of operon *yfc*OPQRSUV, three genes of operon *yra*HIJK and three genes of operon *elf*ADCG (*ycb*QRST) increased more than fourfold and three genes of operon *yehDCBA* increased severalfold.

THz radiation raises fivefold the expression of all seven genes of the *yad* operon: *yadN* (major subunit), *htrE* (chaperone), *yadMLK* (minor subunits), *yadC* (adhesive tip) and *ecpD* (fimbrial chaperone)^33^. The *yad* operon reacts to changes in temperature, dissolved oxygen content of the medium, and other environmental factors^34^. Constitutive expression of cryptic operon *yad* is also known to give rise to biofilms and various adherent structures visible under a microscope; therefore, its expression may be crucial for the adhesion process, thereby initiating the aggregation of irradiated cells, and according to electron microscopy, resulting in the emergence of unusual aggregates consisting of 7 nm bundled pili*.*

THz radiation induced the genes of Curli-type amyloid fimbriae that belong to two operons: *csgBA* and *scgDEFG*^35^. It is reported that genes *csgA* encoding the structural protein of amyloid fimbriae are under the control of fimbrial adhesin YadC^36^.

Additionally, the THz radiation induced genes underlying the biosynthesis of colanic acid, not synthesized in *E. coli* under the normal conditions of planktonic growth. Biosynthesis of colanic acid is induced by damage to cell envelope structure, by osmotic shock, lowered culture temperature, or changes in the structure of the lipopolysaccharide matrix^37–39^. Colanic acid forms a negatively charged polysaccharide capsule around cells of *E. coli*, *Salmonella*, and other gamma-proteobacteria during the transition from a planktonic to adherent state^40^. At least two metabolic pathways and more than 20 genes are involved in colanic acid biosynthesis^40^, among them, 17 genes are upregulated by THz irradiation.

According to our data, the irradiation causes aggregation of bacterial cells not only with the participation of pili. After irradiation bacteria was in contact through side surfaces along their long axis, and there are cases of adhesion between bacterial cells via their apical regions. Similar changes along with asymmetric formation of the septum, agglutination of fimbriae, and adhesion between bacteria have been described in studies on uropathogenic bacteria *Streptococcus pneumoniae*^41^ and *E. coli*^42^. It is known that gram-negative bacteria synthesize nonfimbrial adhesins, which ensure tight contact between bacteria and a substrate. This is especially true for pathogenic strains of *E. coli*^43^.

## Conclusion

By transcriptomic methods (RNA-seq), differential expression of the *E. coli* genome was investigated after THz irradiation, and upregulation of 546 genes more than fourfold and downregulation of 195 genes more than twofold were documented. Among the upregulated genes in the irradiated culture samples, there are genes governing the adhesion and aggregation of *E. coli* cells and inhibitors of cell division. Furthermore, we identified the genes downregulated under THz irradiation that control *E. coli* cell division*.* As a consequence, the processes of adhesion between cells take place, multiple agglutination of fimbriae proceeds, and cell division is blocked.

Electron microscopic examination of the irradiated cells uncovered mutual association of pili into “bundle-like” structures and confirmed the results of the bioinformatics analysis of the RNA-seq data: irradiated bacteria come into multiple extensive contacts involving or not involving pili. Most bacteria are in contact through side surfaces along their long axis, but there are cases of adhesion between bacterial cells via their apical regions. In control (unirradiated) samples, *E. coli* cells as a rule do not aggregate into large conglomerates and do not manifest the signs of their tight direct contacts, even though some bacteria are connected through sex F pili. By methods of electron microscopy, in the irradiated culture, we registered the emergence of defective invagination of the bacterial envelope at the beginning of cell division, in good agreement with the results from the bioinformatics analysis of the pool of synthesized RNAs.

## Methods

### Irradiation of cells

For the treatment of *E. coli* cells with THz radiation, the Novosibirsk Free Electron Laser (NovoFEL) facility was used, which is a part of a multi-access center (the Siberian Synchrotron and Terahertz Radiation Centre; Budker Institute of Nuclear Physics SB RAS, Novosibirsk, Russia). NovoFEL settings are listed in Table 3^44^.

**Table 3:** Parameters of irradiation by NovoFEL.

Parameter	Value
Frequency, THz/wavelength, µm	1.25–3.75/240–80
Pulse repetition frequency, MHz	5.6–22.4
Pulse duration, ps	40–100
Average power, W	Up to 500
Peak power, MW	Up to 0.8
Minimum linewidth, %	0.2

In these experiments, a classic research object of molecular biology was employed: *E. coli* cells (laboratory strain *E. coli* K12 JM109). For this work, a specially equipped biological workstation was used at the Siberian Synchrotron and Terahertz Radiation Centre. The overall workflow of cultured-cell irradiation was similar to the one we have described earlier^5,7^. For uniform irradiation of the volume of *E. coli* cultures, they were irradiated in a special cuvette^6^.

To carry out reproducible experiments, aliquots of a cell culture in the middle of the logarithmic growth phase in the LB medium (10 g/L Bacto Trypton, 10 g/L NaCl, 5 g/L yeast extract, pH 8.0; 100 μg/mL ampicillin) were frozen beforehand in 50% glycerol. Before the experiment, precultures were prepared from the frozen aliquots by culturing for 16 h in LB; from this suspension, new cell cultures were then started via inoculation, and when OD_600_ reached 0.8 (incubation in the thermoshaker at 37 °C for ~ 3 h), the cells were exposed to THz radiation. The cells were irradiated for 15 min with constant temperature control within 37 ± 2 °C in 50 μL suspensions in a special cuvette; the radiation had a wavelength of 130 μm and a power density of 1.4 W/cm^2^. In parallel, control cells were placed in an identical cuvette for 15 min incubation in a thermostat at 37 °C; further manipulations with the irradiated and nonirradiated cells were performed in the same way. For RNA-seq analysis, after the above-mentioned 15 min irradiation or incubation, to develop a response, the cells were collected from cuvettes, kept at 37 °C for 10 min and then were pelleted by centrifugation for 2 min at 1000×*g*; the supernatant was discarded, and the cell pellet was frozen in liquid nitrogen. To obtain a sufficient amount of material for RNA-seq analysis, the samples of the experimental and control cells were pooled pairwise; the experiment was conducted in duplicate.

### RNA-seq

RNA isolation from 100 μL of an *E. coli* culture was performed by means of the PureLink RNA Mini Kit (Ambion) according to the manufacturer’s instructions. To remove ribosomal RNA sequences, the Ribominus Transcriptome Isolation Kit (Bacteria) was employed. Concentration of the purified RNA was measured on a Qubit fluorometer with the RNA High Sensitivity Kit, and RNA quality was evaluated using Bioanalyzer 2100 with RNAPico kits. Libraries for the sequencing were prepared with the TruSeq Stranded mRNA Library Prep Kit (Illumina). The sequencing was carried out on a NextSeq instrument (Illumina) with the NextSeq^®^ 500/550 High Output Kit v2 (Illumina) (75 cycles). The library preparation and sequencing were conducted at the Interinstitutional Shared Center “Genomics” (Institute of Cytology and Genetics, SB RAS).

The quality of the obtained raw Fastq files was checked and analyzed with FastQC. To improve the quality of the raw reads we employed the Trimmomatic tool^45^ using these procedures: removing a base from either the start or end position if the quality was low; trimming bases on a sliding window method; removing any remaining reads that are < 36 bases long. The trimmed reads were aligned to the annotated *E.coli* genome as retrieved from the Ensembl database (June 2008, assembly ASM1942v1). Alignment was performed using TopHat2^46^. The alignments were post-processed into sorted BAM files with SAMTools version 1.4^47^. Reads were attributed to genes using the htseq-count tool from the “HTSeq” framework version 0.7.2^48^ based on gtf files with coordinates of genes from ASM1942v1and indexed SAM file. Differential expression analysis was performed with DESeq2^49^.

### Electron microscopy

*Escherichia coli* cells after the THz irradiation (or control sample) were collected into a microfuge tube, incubated for 10 min at 37 °C and then were used for the preparation of microscopy samples. For this purpose, 300 mesh grids were utilized with a formvar film that was covered by a thin layer of carbon film. The grids were deionized in a plasma cloud on a sputtering device, thereby ensuring better adhesion of the bacteria to the film. Onto a grid with a film fixed by tweezer, 4 µL of a sample was applied and kept there for 30 s. After blotting of excess liquid with filter paper, further processing of the samples was conducted in drops (20 µL) of solutions placed on parafilm. First, a sample was fixed in a drop of 2.5% glutaraldehyde in 0.1 M phosphate buffer (pH 7.4) for 3 min, then the fixative was washed off the grid sequentially in two drops of 0.1 M phosphate buffer, with the removal of excess liquid each time. After that, contrast was enhanced in a drop of a 0.5% aqueous solution of uranyl acetate for 3 min, followed by drying. Three independent identical experiments were conducted, with five samples (biological replicates) on grids in each. The samples were visualized by means of a transmission electron microscope, JEOL-1400 (JEOL, Japan), with a Veleta camera (Olympus, USA) and iTEM 5.1 software (Olympus, USA).

For a comparative quantitative analysis of the numbers of bacterial cells with pili, the numbers of double, triple, and quadruple (clumped) pili per cell, and the number of dividing cells, 50 *E. coli* cells (in each control and experimental sample) were examined that were randomly distributed on films in different areas of three mesh grids. The analysis was carried out as two independent experiments.

## Supplementary Information

Supplementary Information 1.

Supplementary Information 2.

## Data Availability

The sequencing data generated during this study have been deposited to the Sequence Read Archive (SRA), under accession PRJNA648263.
