# Supplementary material for: E. coli aggregation and impaired cell division after terahertz irradiation
Source: Sci Rep. 2021 Oct 14;11:20464. doi: 10.1038/s41598-021-99665-3 (PMC8516962; doi:10.1038/s41598-021-99665-3)
Supplement: Supplementary file 1 — Supplementary Information 1. [file 41598_2021_99665_MOESM1_ESM.pdf]

Supplementary Table S1 Functional enrichment data on the set of *E. coli* genes upregulated by the THz irradiation, with relations identified via the STRING Web service.

pathway ID	description	false discovery rate	matching proteins in network (labels)
**Biological Process**			
GO.0043711	pilus organization	4.63E-09	elfC, elfD, htrE, sfmC, sfmD, yadV, ybgP, ybgQ, yehB, yehC, yfcS, yhcA, yhcD, yqiH, yraI, yraJ
GO.0007155	cell adhesion	2.49E-07	csgA, csgB, elfA, pgaC, sfmA, sfmF, sfmH, yadC, yadK, yadL, yadM, yadN, ybgD, ybgO, ydeQ, ydeR, yfcQ, yfcR, yfcV, yqiI, yraH
GO.0015628	protein secretion by the type II secretion system	3.75E-06	gspC, gspD, gspE, gspF, gspG, gspH, gspI, gspJ
GO.0030030	cell projection organization	7.24E-05	elfC, elfD, fliP, fliR, htrE, sfmC, sfmD, yadV, ybgP, ybgQ, yehB, yehC, yfcS, yhcA, yhcD, yqiH, yraI, yraJ
GO.0009297	pilus assembly	0.00665	elfC, htrE, sfmD, ybgQ, yehB, yhcD, yraJ
GO.0009306	protein secretion	0.00938	fliP, gspC, gspD, gspE, gspF, gspG, gspH, gspI, gspJ, gspK
**Molecular Function**			
GO.0015288	porin activity	1.29E-06	bglH, elfC, htrE, nanC, ompG, ompL, ompN, phoE, sfmD, uidC, wza, ybgQ, yehB, yhcD, yraJ
GO.0044183	protein binding involved in protein folding	0.00023	elfD, sfmC, yadV, ybgP, yehC, yfcS, yhcA, yqiH, yraI
GO.0015473	fimbrial usher porin activity	0.000285	elfC, htrE, sfmD, ybgQ, yehB, yhcD, yraJ
**Cellular Component**			
GO.0009289	pilus	3.52E-10	csgA, csgB, elfA, sfmA, sfmF, sfmH, yadC, yadK, yadL, yadM, yadN, yadV, ybgD, ybgO, ydeQ, ydeR, yfcQ, yfcR, yfcV, yqiI, yraH
GO.0042995	cell projection	4.44E-08	csgA, csgB, elfA, fliE, fliP, fliR, sfmA, sfmF, sfmH, yadC, yadK, yadL, yadM, yadN, yadV, ybgD, ybgO, ydeQ, ydeR, yfcQ, yfcR, yfcV, yqiI, yraH
GO.0015627	type II protein secretion system complex	5.15E-07	gspC, gspD, gspE, gspF, gspG, gspH, gspI, gspJ
GO.0046930	pore complex	0.00129	bglH, nanC, ompG, ompL, ompN, phoE, uidC, wza

Supplementary Table S2 Functional enrichment with GO terms in the set of *E. coli* genes downregulated by the THz irradiation. The relations were identified using the STRING Web service.

pathway ID	pathway description	false discovery rate	matching proteins in network (labels)
**Cellular Component (GO)**			
GO.0044444	cytoplasmic part	3.95e-15	accB,accD,ansB,aspA,aspC,crr,deoD,fumA,gapA,glmM,glpA,glpK,gltA,katG,lpdA,lysU,maeB,nusA,ppiB,ptsH,ptsI,purA,raiA,rihC,rpiA,rplA,rplC,rplI,rplL,rplO,rplR,rplT,rpmC,rpmD,rpmE,rpmI,rpmJ,rpsA,rpsD,rpsE,rpsG,rpsH,rpsI,rpsJ,rpsK,rpsR,serS,skp,sodB,tnaA,tpiA,ubiC
GO.0032991	macromolecular complex	7.19e-14	accB,accD,atpC,cydB,cyoA,cyoB,cyoC,cyoD,dppA,dppB,dppC,eno,glpA,hupA,nuoB,nuoE,nuoG,nuoH,nuoI,nuoJ,nuoL,nuoM,oppA,par,ptsH,ptsI,raiA,rplA,rplC,rplI,rplL,rplO,rplR,rplT,rpmC,rpmD,rpmE,rpmI,rpmJ,rpsA,rpsD,rpsE,rpsG,rpsH,rpsI,rpsJ,rpsK,rpsR,sdhA,sdhC,secE,xseB,yajC
GO.0005840	ribosome	1.45e-13	raiA,rplA,rplC,rplI,rplL,rplO,rplR,rplT,rpmC,rpmD,rpmE,rpmI,rpmJ,rpsA,rpsD,rpsE,rpsG,rpsH,rpsI,rpsJ,rpsK,rpsR
GO.0070469	respiratory chain	7.5e-12	cyoA,cyoB,nuoB,nuoE,nuoG,nuoH,nuoI,nuoJ,nuoL,nuoM,sdhA,sdhC
**Biological Process (GO)**			
GO.0006091	generation of precursor metabolites and energy	1.33e-10	aspA,cydB,cydX,cyoA,cyoB,cyoC,cyoD,eno,fda,frdA,fumA,gapA,gldA,glpC,gltA,gpmA,lpdA,nuoB,nuoE,nuoG,nuoH,nuoI,nuoL,nuoM,pflB,pgk,sdhA,sdhB,sdhC,sdhD,tpiA
GO.0009060	aerobic respiration	4.2e-09	aspA,cydB,cyoA,cyoB,cyoC,cyoD,fumA,gltA,nuoB,nuoG,nuoH,nuoI,nuoM,sdhA,sdhB,sdhC,sdhD
GO.0006412	translation	3.15e-10	asnA,ettA,fus,lysU,rplA,rplC,rplI,rplL,rplO,rplR,rplT,rpmC,rpmD,rpmE,rpmI,rpmJ,rpsA,rpsD,rpsE,rpsG,rpsH,rpsI,rpsJ,rpsK,rpsR,serS,tufA
GO.0045333	cellular respiration	5.78e-07	aspA,cydB,cyoA,cyoB,cyoC,cyoD,frdA,fumA,gltA,nuoB,nuoE,nuoG,nuoH,nuoI,nuoL,nuoM,pflB,sdhA,sdhB,sdhC,sdhD
**Molecular Function (GO)**			
GO.0003735	structural constituent of ribosome	2.56e-11	rplA,rplC,rplI,rplL,rplO,rplR,rplT,rpmC,rpmD,rpmE,rpmI,rpmJ,rpsA,rpsD,rpsE,rpsG,rpsH,rpsI,rpsJ,rpsK,rpsR
GO.0048038	quinone binding	7.05e-07	cyoA,cyoB,nuoB,nuoE,nuoG,nuoH,nuoI,nuoJ,nuoL,nuoM,sdhC
GO.0003723	RNA binding	8.94e-07	accD,cspA,cspC,cspD,ettA,fus,nusA,rplA,rplC,rplI,rplO,rplR,rplT,rpmC,rpmE,rpsA,rpsD,rpsE,rpsG,rpsH,rpsI,rpsJ,rpsK,rpsR,rsuA,smpB,tufA

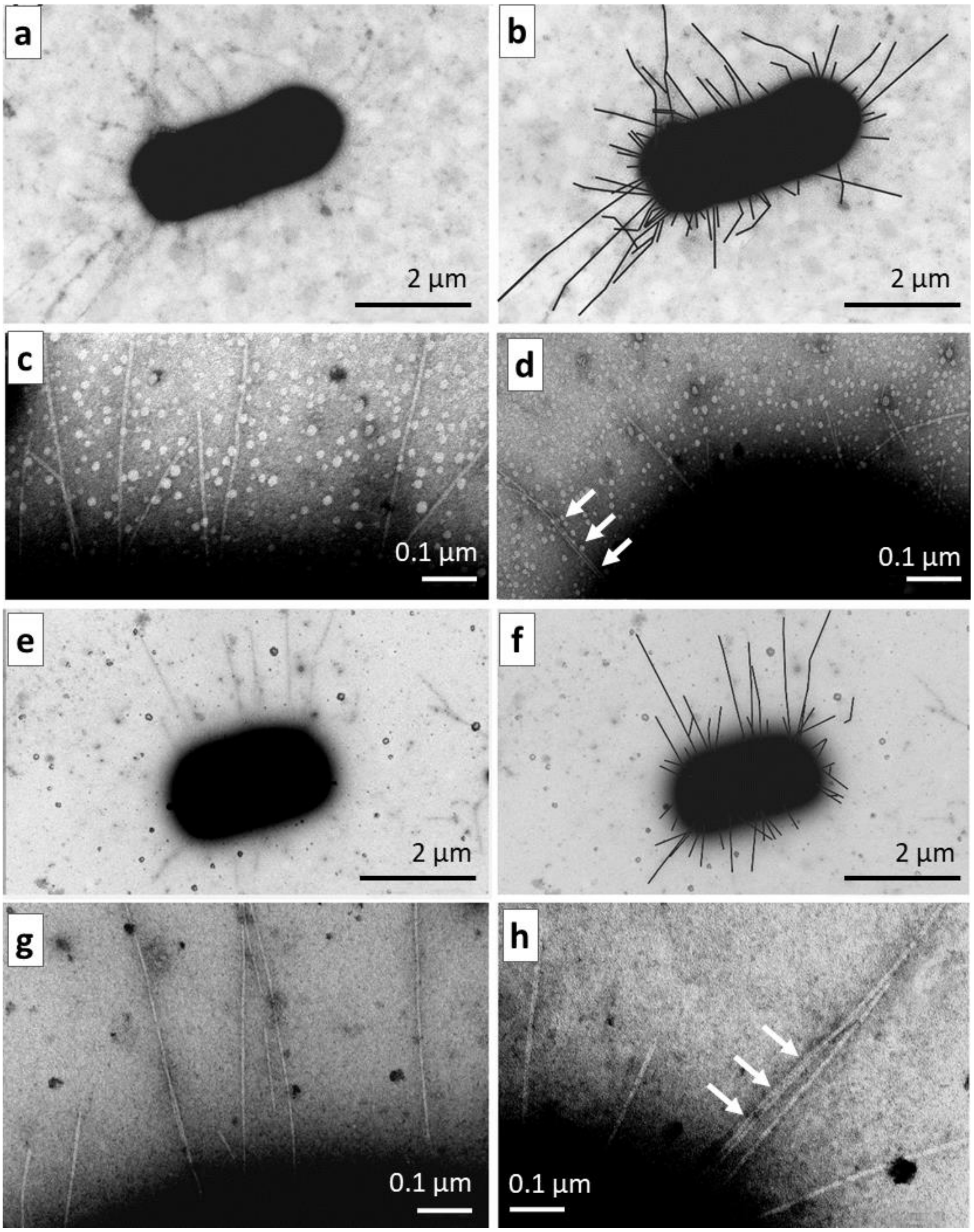

Supplementary Figure S3. View of *E. coli* cells with type 1 pili in a control sample. (**a, e)** Snapshots of two bacterial cells at low magnification. (**b, f)** Copies of the above snapshots with pili indicated by the black color. (**c, d**) Pili in magnified parts of panel a; in panel d, light arrows point to a two-pilus bundle. (**g-h**) Pili in magnified parts of panel e; in panel h, the black arrows indicate two-pilus bundles. The scale bar in panels a, b, e, and f is 2 µm, and in panels c, d, g, and h is 0.1 µm.

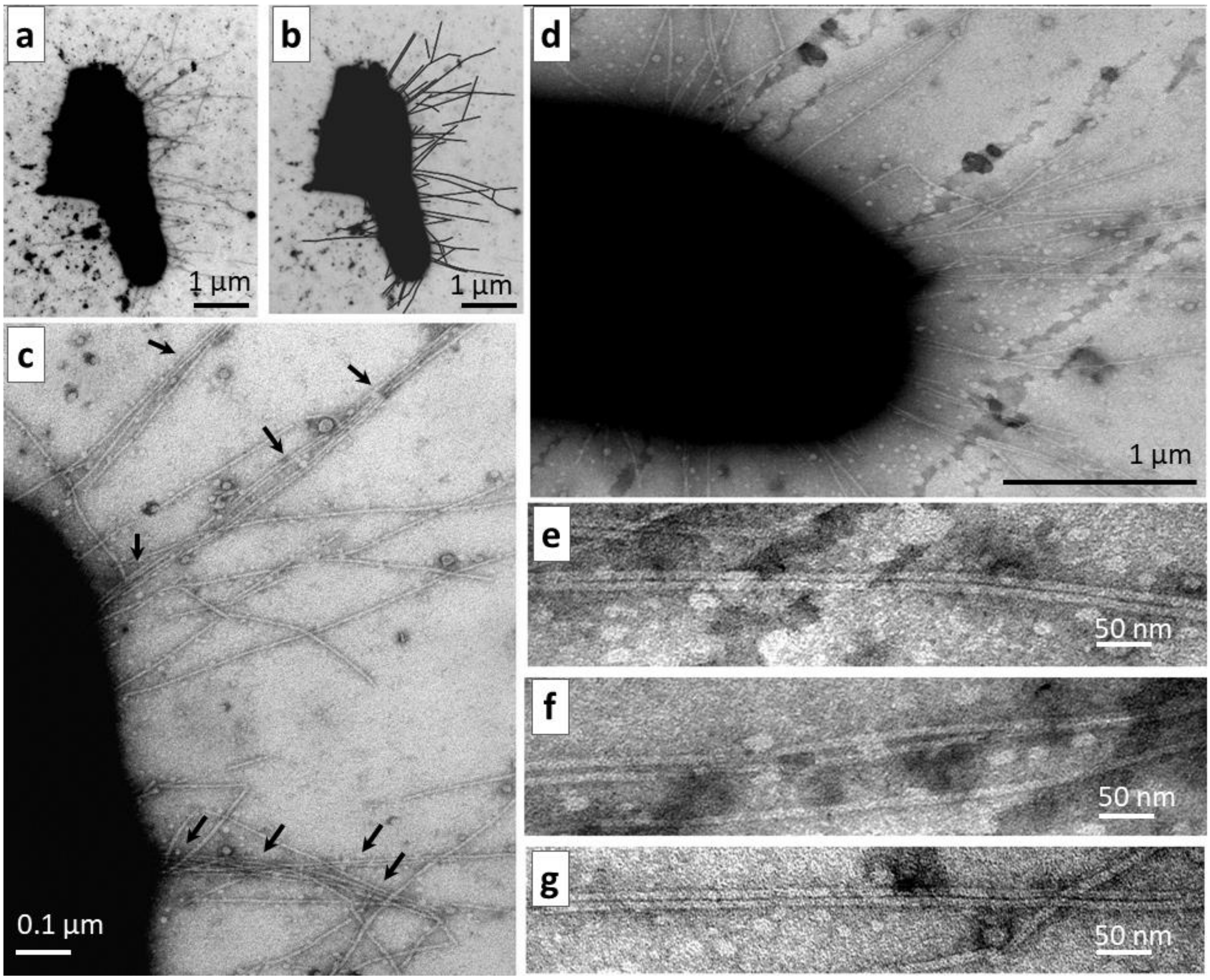

Supplementary Figure S4. Specific features of bundling of type 1 pili on *E. coli* cells after the THz irradiation. (**a**) Two aggregated bacterial cells, one of which carries numerous type 1 pili. (**b**) The copy of panel a in which pili are indicated with the black color. (**c, d**) Magnified part of panel a. Two-pilus bundles (indicated by arrows) are seen both near the surface of the outer membrane of a bacterium and at various distances from it. (**e–g**) Examples of two-pilus bundles. The scale bar is 1 µm in panels a, b, and d; 0.1 µm in c; and 50 nm in panels e–g.
